# A geminiviral amplicon (VA) derived from *Tomato leaf curl virus *(ToLCV) can replicate in a wide variety of plant species and also acts as a VIGS vector

**DOI:** 10.1186/1743-422X-6-152

**Published:** 2009-09-29

**Authors:** Prerna Pandey, Nirupam R Choudhury, Sunil K Mukherjee

**Affiliations:** 1 Plant Molecular Biology Group, International Centre for Genetic Engineering and Biotechnology (ICGEB), Aruna Asaf Ali Marg, New Delhi - 110 067, India

## Abstract

**Background:**

The *Tomato leaf curl virus *(ToLCV) belongs to the genus *begomoviridae *of the family *Geminiviridae*. The 2.7 kb DNA genome of the virus encodes all the information required for viral DNA replication, transcription and transmission across the plant cells. However, all of the genome sequences are not required for viral DNA replication. We attempted to reveal the minimal essential region required for DNA replication and stable maintenance. The phenomenon of Virus Induced Gene Silencing (VIGS) has recently been observed with several geminiviruses. We investigated whether the minimal replicating region was also capable of producing siRNAs *in planta* and a VIGS vector could be constructed using the same minimal sequences.

**Results:**

We have constructed vectors containing various truncated portions of the *Tomato leaf curl virus *(ToLCV) genome and established that a segment spanning from common region (CR) to AC3 (ORF coding for a replication enhancer) was the minimal portion which could efficiently replicate in a variety of both monocot and dicot plants. A viral amplicon (VA) vector was constructed using this region that produced siRNAs from various sites of the vector, in a temporal manner in plants, and hence can be used as a VIGS vector. The tomato endogene PCNA was silenced using this vector. Introduction of a mutation in the ORF AC2 (a silencing suppressor) increased the silencing efficiency of the newly constructed vector several folds.

**Conclusion:**

Our study reveals that the vector is capable of replicating in diverse plant species and is highly efficient in silencing endogenes like *PCNA *of the host plant, thus acting as a VIGS vector. We observed that the geminiviral ORF AC2 functioned as a silencing suppressor and a null mutation in this ORF increased the efficiency of silencing several fold. This is the first report of construction of improved VIGS vector by mutation of the resident silencing suppressor gene. The present study opens up the possibility of using such VIGS vectors in silencing the host genes in a broad range of plant hosts.

## Background

*Tomato leaf curl virus *(ToLCV) is a member of *Geminiviridae *family (*Begomovirus *genus) and is transmitted through white fly (*Bemisia tabaci*) to infect tomato (*Lycopersicon esculentum*) plants of all known cultivars. Such infection causes stunted growth and leaf curling in the plants leading to a great reduction in the crop yield. ToLCV is now being recognized as one of the most important threats to tomato crop in both tropical and subtropical parts of the world. Although tomato is its natural host, ToLCV is known to infect other *Solanaceous *species as well, thereby adding to its devastating effects. Out of all the varieties available for *Tomato leaf curl virus*, the ToLCV (New Delhi variety) is most abundant in this part of north India and is mainly responsible for destruction of tomato and other crop plants in our country.

The organization of ToLCV genome is similar to the DNA-A component of the viral genomes of most other members of the *Begomovirus *genus. The ToLCV genome contains an intergenic region of about 200 bp from where the RCR initiates and this region harbors a few repeat sequence elements that are occupied by the main replication initiation protein named as Rep or AC1 during RCR. This initiation zone is also known as the common region (CR). Besides Rep protein, the DNA-A also encodes for only few ORFs, viz., AC2, AC3, AC4, AV1 and AV2, which share a certain amount of homology with the corresponding ORFs encoded by other begomoviruses. The replication of viral DNA is initiated by creation of a nick by Rep at the conserved nonameric sequence TAATATT↓AC within the CR region. The 3'-OH end thus created is then extended by host polymerase(s) which, in turn, is aided by the viral Rep and other host proteins to generate the viral ssDNA genome. The ssDNA genomes are subsequently encapsidated by coat proteins (encoded by AV1) to produce virions. AC3 is known to function as a replication enhancer (REn) and is required for efficient replication of its genome, while both AC2 and AC4 have been implicated in suppression of the gene silencing phenomena.

**Figure 1:**
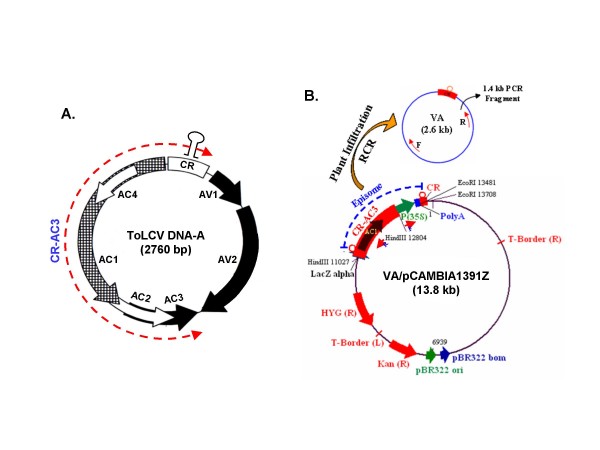
**Genome organizations of ToLCV and the viral amplicon (VA)**. (A) The genome organization of ToLCV (New Delhi isolate) based on its nucleotide sequence [Genbank:DQ629101] where the region spanning from CR to AC3, used for further manipulations, is marked (red hyphenated arrow). The locations of various ORFs are marked by thick arrows. The stem-loop structure assumed by a part of common region (CR) is indicated on the top of CR-region. (B) The map of the viral amplicon cloned in pCAMBIA1391Z vector backbone (designated as VA/pCAMBIA1391Z), where the positions of the cloned fragments and of the various restriction sites are shown. The 2.6 kb episome spanning the region CR to *AC3 *and another CR, released through RCR on infiltration of the plants by above plasmid is schematized. The internal primers (F and R) used to check the formation of episome, are shown as black arrows within the episome.

To study the processes of replication of ToLCV DNA, it is imperative to map the minimal replicon of the viral DNA. This region might include the origin of RCR in a mandatory manner along with Rep (or AC1) plus other regions that encode factors to serve the accessory role(s) in DNA replication. Earlier, we had established a yeast model of geminiviral DNA replication [1] and using the model with various truncations of the viral DNA, we mapped the replicon region. As shown in Fig. 1A, the minimal replicon turned out to be the CR-AC3 segment and the CR along with the AC1 component of this segment was the most vital region. Mutation and deletion in the AC3 component allowed replication but in an irreproducible and severely damped manner [2]. Hence we took the CR-AC3 region as the replicon of the ToLCV DNA. A similar region was also found to act as a replicon of the DNA-A component of the geminivirus, MYMIV [3].

Virus DNA replication and its consequent growth *in planta *is also determined by the interaction of host RNAi factors with the viral proteins, especially the suppressors of RNAi. The geminiviruses have been shown to elicit gene silencing through RNAi (RNA interference) mechanism. Following infection of the hosts by the plant viruses including geminiviruses, long dsRNAs are generated either as replication or transcription intermediates that establish the viral RNA silencing mechanism in the host. This is one of the major ways used by plants as a defense mechanism against the infecting viruses. This mechanism has allowed the development of an important tool to silence endogenous plant genes and these tools are often called as VIGS vectors [4].

Such VIGS vectors transiently suppress the expression of the gene through degradation of transcripts, without altering the gene itself. Thus, in sharp contrast to conventional knock-out mutagenesis techniques, the VIGS vectors allow the study of genes that would normally lead to lethality when disrupted. Since the discovery that virus derived vectors can be effectively used for silencing genes in plant tissues [5], a number of studies have been carried out in an attempt to silence various plant genes using geminivirus genome based vectors [6-9]. Such studies indicate that the VIGS vectors, constructed based on geminiviral genome, can mostly be used with considerable success to silence host genes. However, whether such phenomenon is conserved amongst all other members or such phenomena could be regulated for enhanced efficiency etc., are matters of intense investigation.

Till date the genome sequencing of various plant species has resulted in the identification of a large number of open reading frames (ORFs). Elucidation of the functions of all these ORFs poses an immediate challenge and necessitates the development of quick and reliable methods to study the functional genomics. T-DNA insertion [10] and transposon based [11,12] methods have been used for such analyses. However these methods are associated with a large number of technical limitations like difficulty in tagging all genes, lack of phenotype due to high degree of gene duplication in the plant genome, lethality caused by insertions, etc. VIGS offers an attractive and quick technique to arrest expression of a gene without needing to transform genetically. As the effects of silencing are not permanent, the functional studies can be carried out without causing any serious damage to the plant. VIGS methodology has found various successful applications such as molecular farming and functional gene characterization in tobacco [13], *Arabidopsis thaliana *[14,15], tomato [16], pepper [17], potato [18], legume [19], cassava [6] and Mungbean [20], etc. In addition, the use of VIGS has the advantage that the time required for phenotypic analysis is considerably shorter.

Transmission of geminiviruses by whitefly (*Bemisia tabaci*) requires coat protein [21,22]. However, constructs containing whole or part of the geminivirus genome can be infiltrated into the plants by various methods under laboratory conditions. Out of the few available methods, viz., gene gun bombardment method [23], rubbing DNA onto carborundum dusted leaves [24], *Agrobacterium tumefaciens *mediated agroinoculation [25], etc. *Agrobacterium *mediated transfer offers a relatively convenient and reproducible tool to infect plants with geminiviruses. Using this technique, either the whole or part of the gemini-genome could be used to study the processes of DNA replication and associated silencing.

In our present study, we report the development of a construct containing a region spanning from CR to AC3 of ToLCV (New Delhi) genome that has been cloned in a binary vector pCAMBIA1391Z. We demonstrate that this vector could be introduced in various plant systems and the introduction leads to formation of an episomal circle, called as a Viral Amplicon (VA). The episome is formed following the RCR release of a suitable fragment of the viral genome that acts as the viral amplicon (Fig. 1B). The viral amplicon was found to be generated and replicated in plants like tomato, tobacco (*Nicotiana xanthii*), *Arabidopsis thaliana *and rice (*Oryza sativa*, variety Pusa Basmati). We also show that the mutation in either *AC2 *or *AC4 *gene, present within VA, considerably reduces the efficiency of replication of VIGS vector in tomato plants, while mutations in both *AC2 *and *AC4 *drastically reduce the replication efficiency. We further investigated whether such a viral amplicon could be used as a virus induced gene silencing (VIGS) vector and demonstrated its efficacy in silencing endogenous PCNA gene of tomato. An introduction of the null mutation in the AC2 ORF of the VIGS leads to enhanced silencing of PCNA, thus increasing the efficiency of the VIGS vector.

## Results and Discussion

### Construction of Viral Amplicon (VA) from ToLCV DNA-A

The map of ToLCV (New Delhi isolate) genome, based on its nucleotide sequence [GenBank:DQ629101], is presented in Fig. 1A, where the region spanning from CR to AC3, used for further manipulations, is marked (hyphenated red arrow). The strategy adopted to construct viral amplicon (VA) has been described in detail in the "Methods" section. The orientations of the fragments cloned at different steps were checked by restriction digestion of DNA and by PCR using appropriate primers. The final resulting plasmid, designated as VA/pCAMBIA1391Z, is schematized in Fig. 1B showing the various restriction sites and the positions of the cloned fragments. Whenever this plasmid is delivered in the leaves of the plants via agroinoculation, a circular episome (marked VA) gets excised out and replicates independently in leaves for a period of time. The episome formation and its replication can be detected by PCR using a set of divergent primers, shown as red arrows in Fig. 1B, or by Southern blotting [3,26].

### Versatility of replication of VA

The VA/pCAMBIA1391Z construct was introduced into the leaves of tomato, tobacco, *Arabidopsis *and rice plants by using *Agrobacterium *(strain LBA4404) mediated agroinfiltration technique to check for the replication efficiency of the construct. Equal titre of the culture was used for inoculation in all the cases. Inoculated leaves were collected at various intervals of time post-infiltration and total DNA was isolated from these samples as described ("Methods" section) for Southern blotting or PCR amplification.

**Figure 2:**
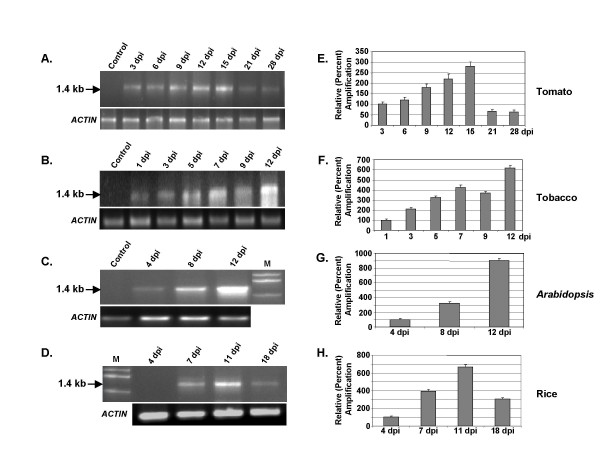
**The viral amplicon construct, VA/pCAMBIA1391Z, replicates in both dicot and monocot plants**. The plants (Tomato, Tobacco, *Arabidopsis *and Rice) were agro-infiltrated with this construct, and the release of episome was checked by PCR using internal primers. The amplification products (1.4 kb) were separated on 1% agarose gel and are presented in panels A, B, C and D for Tomato, Tobacco, *Arabidopsis *and Rice respectively. The band intensities were quantified and the normalized values with respect to corresponding loading controls (*ACTIN*) are plotted as bar graphs in panels E, F, G and H respectively. In these panels the respective values at 3, 1, 4 and 4 dpi were arbitrarily assigned as 100%. The standard deviations shown are based on three independent experiments.

As the CR-AC3 segment was derived from ToLCV, we examined the episome formation in tomato plants first. The predicted sizes of the VA episome and its PCR-amplified product with the divergent primers are 2.6 kb and 1.4 kb respectively. The PCR reactions were carried out with the isolated genomic DNA as mentioned and the parallel reactions were also carried out with the actin gene primers for internal loading controls as shown in Fig. 2A. To exclude the contribution of the unreplicated input DNA towards the amplified products, the isolated genomic DNAs were digested with *Dpn*I before carrying out the PCR. The results revealed that the episome accumulation reached a peak at 15 days post inoculation and subsequently loss of VA accumulation occurred, perhaps reflecting the degradation of the episome. Since tobacco and tomato belong to the same family (*Solanaceae*), VA accumulation was also tested in tobacco plants. The accumulation of VA seemed to be higher in tobacco than in tomato but the overall pattern of accumulation appeared to be similar in both the plants (Fig. 2B and 2F).

Although very few geminiviruses infect *Arabidopsis*, a member of the begomovirus family, viz., *Cabbage leaf curl virus*, is known to infect *Arabidopsis*. Hence we wondered if the VA accumulation also occurs in *Arabidopsis*. Accordingly, the plants were agroinfiltrated with the abovementioned VA/pCAMBIA1391Z DNA constructs and the DNA isolated from the infiltrated plants was examined for the presence of VA circles. Fig. 2C and 2G indicates that *Arabidopsis *was quite efficient in supporting the replication of VA DNA. Further, to test the versatility of VA accumulation, we carried out similar experiments in the model monocot rice plant. It is worthwhile to mention that rice has not been reported as the host plant for the geminiviruses as yet. The results shown in Fig. 2D and 2H clearly indicate that rice also supported VA accumulation quite well. Thus the characteristics of supporting the VA replication seem to be a general feature of most of the plants. The intensities of the 1.4 kb bands, as shown in Fig. 2A-D, were quantified using ImageQuant TL software and were normalized with respect to the corresponding actin controls. The average normalized intensity values (based on three independent studies) are presented as bar graphs in Fig. 2E-H. The gradual decline in the band intensities at longer time interval (>11-15 dpi) is expected in view of the degradation of DNA by the nucleases present in the host plant system.

It is logical to assume that the high level of accumulation of VA resulted due to the RCR mode of replication as the VA DNA contained all the cis elements for replication like the parental viral DNA. Previously, we had demonstrated that similar construct using the CR-AC3 region of *Mungbean yellow mosaic India virus *(MYMIV) was quite efficient in replication in *Saccharomyces cerevisiae *as well as in plants (*Nicotiana xanthi*) [1,3]. Our findings that any further truncation of CR-AC3 region rendered the construct unable to replicate in any of the systems studied above (data not shown) clearly support the proposition that the region CR-AC3 of ToLCV, like that of MYMIV, is the minimal region necessary and sufficient for the replicon activity of the viral amplicon (VA).

### Detection of siRNA

**Figure 3:**
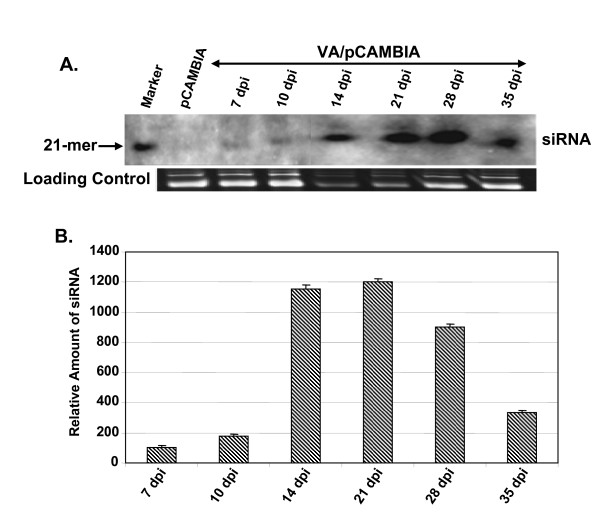
**Northern hybridization showing the time kinetics of siRNAs accumulation in tomato plants agroinfiltrated with VA/pCAMBIA1391Z**. Northern hybridization using CR-AC3 DNA fragment as a probe showing the formation of siRNAs in infiltrated tomato leaves at indicated time points (dpi). A labeled 21-mer oligonucleotide was used as a size marker. (B) The relative intensities of the siRNA bands were estimated and the normalized values with respect to corresponding loading controls are plotted as bar graphs. The value at 7 dpi was arbitrarily assigned as 100%. The standard deviations based on three independent experiments are shown.

**Figure 4:**
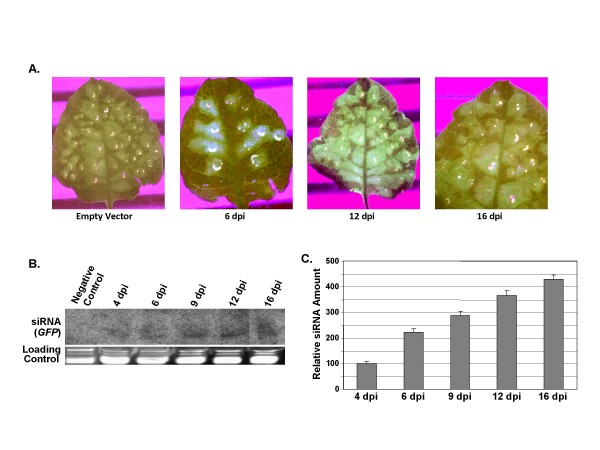
**GFP expression and subsequent siRNA accumulation in tobacco plants as a function of time**. Leaves of tobacco plants infiltrated with full-length *GFP *gene cloned in VA/pCAMBIA1391Z (referred to as VIGS-*GFP*) and with VA/pCAMBIA1391Z (empty vector) were photographed under UV illumination to monitor the expression of GFP at indicated dpi. (B) Autoradiograph showing the formation of siRNAs probed with radiolabeled *GFP *gene. (C) The relative intensities of the siRNA bands were estimated and the normalized values with respect to corresponding loading controls are plotted as a bar graph. The value at 4 dpi was arbitrarily assigned as 100%. The standard deviations shown are based on three independent experiments.

In view of the reported gene silencing phenomena induced by various geminiviruses, we wondered if the ToLCV viral amplicon (VA) was also capable of eliciting any gene silencing in the host. For the purpose, the tomato leaves were agroinfiltrated with the VA/pCAMBIA1391Z construct and the leaves were collected at different time intervals (7 to 35 dpi) and total RNA was isolated from these samples. The level of siRNA in the extracted RNA was ascertained by Northern hybridization using CR-AC3 region of ToLCV as the probe. The autoradiogram shown in Fig. 3A demonstrates that ~21 nt long siRNAs were indeed formed in tomato. The band intensities were quantified and were normalized with respect to respective loading controls. The normalized values of the band intensity, setting arbitrarily the value obtained for 7 dpi as 100%, are represented as bar graph in Fig. 3B. It is clear from the results that the levels of accumulation of siRNA were temporal in nature. The siRNA increasingly accumulated until ~21 dpi, beyond which the levels decreased gradually till 35 dpi. The fact that the siRNAs were available over such an extended period of time might reflect the stability of the small RNA within the host plant. The siRNAs were specific for the viral sequences only as no siRNA could be observed when the vector backbone of pCAMBIA1391Z was used as the probe (data not shown). It is thus apparent that the siRNAs were generated only from the VA episome. To drive the point further, the *GFP *gene of ~0.7 kb was cloned in the MCS site of VA/pCAMBIA1391Z (located downstream of 35S promoter but upstream of the polyA site; Fig. 1B). Following introduction of this plasmid in tobacco leaves, PCR was carried out with the extracted plant genomic DNA materials and the same primers as shown in Fig. 1B. The resulting amplified band was of 2.1 kb size, reflecting the increase of the episome size due to inclusion of the *GFP *gene (data not presented). The expression levels of GFP were monitored at various time intervals (4-16 dpi) by observing the GFP fluorescence of the agro-inoculated tobacco leaves under UV (Fig. 4A). The results indicate that the GFP expression levels increased till around 12 dpi, beyond which the level decreased gradually. The episome accumulation of VA-*GFP*, GFP-fluorescence and the loss of it, etc. in tobacco was found to be almost similar to those in tomato (data not shown). The formation of *GFP*-siRNA was examined from the total RNA of the agro-inoculated leaves by Northern hybridization using the labeled *GFP *probe (Fig. 4B, C). The kinetics of formation of *GFP*-siRNA in tobacco was similar in nature to the one shown in Fig. 3A. It may be noted that agroinoculation with empty vector (pCAMBIA1391Z) did not give rise to any significant level of *GFP*-siRNA (Fig. 4B, lane 1), further substantiating that the production of siRNAs was specific for the episome. Thus the *in planta *synthesis of viral siRNA did not require the presence of the full viral genome.

### Replication efficiencies of wild type and mutated VA constructs in tomato plants

From the above data (Figs. 3 and 4), it is apparent that the viral siRNAs were synthesized during VA accumulation. These siRNAs might limit the accumulation of VA transcripts and consequently inhibit the VA DNA accumulation in turn. However, the VA templates also encode factors like AC2 and AC4, which act as suppressors of siRNAs [27-29]. We have demonstrated earlier that the oversupply of AC2 protein or other RNAi suppressors enhanced replication of VA both in tomato and tobacco [unpublished; 20, 30]. The AC4 protein of begomoviruses has also been shown to act as a silencing suppressor [31]. We argued that the removal of these proteins might negatively impact VA accumulation and hence investigated the effect of null mutation of *AC2 *and *AC4*, both singly and in combination, on the replication efficiencies of VA constructs in tomato plants.

**Figure 5:**
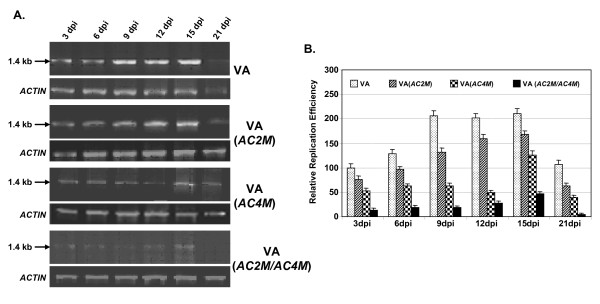
**Comparison of replication efficiencies of both wild type (VA) and various mutants [VA(*AC2M*), VA(*AC4M*) and VA(*AC2MAC4M*)] of VA/ToLCV constructs in tomato plants**. (A) Total DNA was isolated from the tomato leaves agroinfiltrated with VIGS, VA(*AC2M*), VA(*AC4M*) and VA(*AC2MAC4M*) separately and the release of episome at indicated dpi was checked by PCR using internal primers. The respective *ACTIN *controls are shown. (B) The band intensities of the amplified products were quantified and the normalized values with respect to corresponding loading controls (*ACTIN*) are plotted as bar graphs. The corresponding value for VA construct at 3 dpi was arbitrarily assigned as 100%. The standard deviations based on three independent experiments are shown.

We introduced a point mutation in *AC4 *by converting the aminoacid serine at 9^th ^position to a stop codon (TCA → TAA), and this change left the overlapping reading sequence of Rep [CTC(L) → CTA(L)] intact. Similarly the fifth aminoacid of AC2, i.e., Serine was converted to a stop codon [TCA → TAA], leaving the overlapping Rep reading sequence [GTC (V) → GTA (V)] unchanged. The resulting constructs [VA (*AC2M*) and VA (*AC4M*) for mutations in *AC2 *and *AC4 *respectively] were agro-infiltrated into the tomato leaves. Leaves were collected from the plants at various dpi and the replication efficiencies were checked using the PCR technique as mentioned earlier. As is shown in Fig. 5A, the replication with wild type as well as mutant constructs increased till about 15 dpi, beyond which the values decreased in all these cases. To ascertain the changes in a quantitative manner, the band intensities were quantified, normalized with respect to loading controls and plotted as a bar graph (Fig. 5B). For the purpose of comparison, the value for wild type at 3 dpi was arbitrarily assigned as 100%. As is evident from our data, the relative replication efficiencies increased to about 2 fold at 15 dpi and at 21 dpi, the value was reduced to less than that obtained for 3 dpi. It is worth noting that mutation in *AC2 *resulted in a decrease in the replication efficiency by about 25%, while the mutation in *AC4 *caused a more robust decrease (50%).

In order to see if such effects on mutation is synergistic in nature, we created double mutation (in both *AC2 *and *AC4 *genes to generate the VA (*AC2M*/*AC4M*) construct and tested its effects on replication as above. As our data indicate (Fig. 5A, B), the replication efficiency was seriously affected (about 4 fold decrease compared to wild type) on introduction of double mutation. As expected, these evidences established the notion that the removal of the silencing suppressors down regulated the accumulation of VA, perhaps by increasing the biogenesis and function of siRNAs.

### Silencing of PCNA in tomato plants by VA vector

From the data presented above, it appeared that the VA episome could be used as a tool to silence plant endogenes or, in other words, VA could also be used as a VIGS vector. To establish the point, we introduced a portion of tomato PCNA gene in the MCS region of the vector and designated it as VIGS-PCNA (*AC2N*). We hypothesized that this vector would generate PCNA-siRNA in tomato and abolish the PCNA endogene so that the host chromosomal replication and consequent cell division would be abrogated. For the control experiment, we used the VA-*GFP *vector since the *GFP*-siRNA would not affect the tomato replication and cell division.

**Figure 6:**
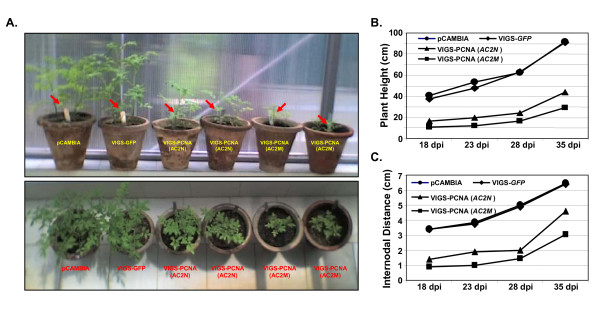
**Mutation of silencing suppressor AC2 leads to altered morphology in tomato plants**. (A) Photograph showing the side and top views of the phenotypes of tomato plants infiltrated with pCAMBIA1391Z (vector backbone), VIGS-*GFP *(negative control), VIGS-PCNA(*AC2N*) and VIGS-PCNA(*AC2M*) as marked, observed at 18 dpi. The red arrows indicate the sites of agroinoculation. The plant heights and the internodal distances of the above infiltrated plants were measured and the values are presented graphically as a function of time (dpi) in panels B and C respectively.

Tomato leaves were agroinfiltrated with these constructs separately or with empty vector as described earlier and the resulting phenotypes were observed over a period of time. The side (top panel) and top (bottom panel) views of the plants are presented in Fig. 6A. As expected, neither empty vector nor VIGS-*GFP *construct caused any noticeable phenotypic change in the plants. Plants infiltrated with VIGS-PCNA(*AC2N*) construct, on the contrary, resulted in a substantial stunting in growth. As is discussed later in this section, the PCNA transcripts were found to be gradually declining over a period of time whereas the levels of PCNA-siRNA increased gradually. Thus the silencing of the PCNA gene, induced by the VA vector, was evidently responsible for the stunting phenotype of the agro-inoculated tomato plants. This phenotype was visible even two months after agro-inoculation.

Despite the appearance of stunted phenotype with the VIGS-PCNA (*AC2N*) construct, some plant growth beyond the site of agro-inoculation (shown by red arrow in Fig. 6A) was observed. Hence we examined if the above-mentioned construct was able to elicit adequate level of PCNA silencing. The CR-AC3 region is also supposed to express the AC2 ORF which is known as an RNAi suppressor [20; Fig. 5]. So we hypothesized that the VA episome, which fails to express the AC2 ORF, might act as a better VIGS vector. In order to validate such assumption, we introduced a point mutation in *AC2 *gene so that the protein translation terminated at the 5^th ^amino acid and the vector bearing the mutation was named as VIGS-PCNA(*AC2M*) as mentioned in the preceding section. When the leaves of tomato plants were agro-inoculated with this mutated vector, the stunting was much more pronounced than that caused by the VIGS-PCNA (*AC2N*) construct. Two (out of total 20) such representative plants are shown in Fig. 6A. From our data, it is clear that the gene silencing effect induced by VIGS-PCNA (*AC2M*) vector is more robust than that caused by the VIGS-PCNA (*AC2N*) vector, thereby supporting our above hypothesis.

In order to analyze plant growths in a more systematic manner, we monitored the plant heights and the internodal distances at regular time intervals. The values are shown in Fig. 6B and 6C respectively in graphical forms. Our data reveal that while the effects of infiltration with empty vector and VIGS-*GFP *constructs on the plant morphology were almost identical, the effects caused by VIGS-PCNA(*AC2N*) were rather drastic. Both the plant height and the internodal distance were greatly reduced (~50 to 65%) on introduction of PCNA, compared to that observed in case of empty vector. The effects were even more pronounced (about 1.5 fold) in case where *AC2 *was mutated. As AC2 is presumed to function as a silencing suppressor, it would be logical to assume that the observed morphological changes in the latter case were caused by enhanced silencing of the PCNA gene in the host.

**Figure 7:**
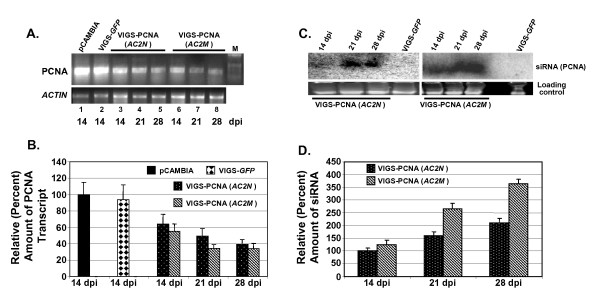
**Mutation of silencing suppressor AC2 leads to increased silencing of *PCNA***. (A) The transcript levels of *PCNA *gene in the agroinfiltrated leaves of tomato plants at various dpi as indicated were estimated through RT-PCR. *ACTIN *gene was used as an internal control. (B) The band intensities were normalized with respect to corresponding controls (*ACTIN*) and are plotted as bar graphs. (C) Autoradiogram showing the levels of siRNAs present in these samples checked by Northern hybridization using PCNA as the probe. (D) The relative intensities of the bands were estimated and the normalized values with respect to corresponding loading controls are plotted as a bar graph. Assignment of the values of 100% in all these panels was arbitrary. The standard deviations shown in the bar graphs (panels B and D) are based on three independent experiments.

We next measured the transcript and the siRNA levels in the infiltrated leaves by Northern blotting as well as by RT-PCR. We isolated total RNA from pCAMBIA1391Z (vector backbone), VIGS-*GFP *(negative control), VIGS-PCNA(*AC2N*) and VIGS-PCNA(*AC2M*) infiltrated plant leaves. We estimated the transcript levels of PCNA by RT-PCR and the results obtained are shown in Fig. 7A. Comparison of the lanes 1 and 2 clearly shows that the PCNA transcript levels were almost identical in the plants infiltrated with either the vector backbone or with VIGS-*GFP*. The plants inoculated with VIGS-PCNA(*AC2N*), on the other hand, had much reduced levels of PCNA transcript level (lanes 3-5). The level was further compromised on agroinfiltration with VIGS-PCNA(*AC2M*) (lanes 6-8). The band intensities were quantified as described earlier and the values normalized with respect to loading controls were plotted as bar graph (Fig. 7B). In constructing the graph, the normalized value for pCAMBIA1391Z was arbitrarily assigned a value of 100%. Our data reflect that the transcript level decreased to ~40% (at 28 dpi) on introduction of tomato PCNA gene in the construct. The transcript level was further reduced (~34% at 28 dpi) when a similar construct with mutated *AC2 *[VIGS-PCNA(*AC2M*)]. These results are in agreement with the notion that the introduction of the VIGS vector harboring a part of an endogenous gene leads to silencing of the gene to a great extent. That such silencing is specific to the gene of interest is discerned from the observation that introduction of any non-homologous gene (*GFP*) did not affect the transcript level to any appreciable extent (94% vis-à-vis 100% for vector backbone).

The above view was further confirmed by directly investigating into the production of siRNAs in such systems. We enriched the small RNA fraction from the total RNA isolated from these plant leaves and separated on a denaturing 15% PAGE. The gel was electro-blotted and probed with radiolabeled PCNA fragment to check for the presence of any gene specific siRNA. The resulting autoradiogram is shown in Fig. 7C. The data clearly indicate the production of siRNA in cases of both VIGS-PCNA(*AC2N*) and VIGS-PCNA(*AC2M*). As is clear from our results, the levels of siRNA were time dependent in nature. We further quantified the band intensities and the normalized values were plotted as a bar graph and presented in Fig. 7D. Analysis of the data reflects that the production of siRNA increased by more than two fold from 14 to 28 dpi in case of VIGS-PCNA(*AC2N*). The siRNA production in case of VIGS-PCNA(*AC2M*) was observed to be considerably higher at all the time points studied. For example, at 28 dpi, the siRNA level was ~1.8 fold higher in case of VIGS-PCNA(*AC2M*) as compared to that for VIGS-PCNA(*AC2N*).

## Conclusion

All these data together clearly established that our newly constructed vector, based on the CR-AC3 region of the geminiviral ToLCV genome, can act as an efficient VIGS vector. The findings further reflect that the ORF AC2 of ToLCV functions as a silencing suppressor and any mutation in this ORF increases the efficacy of the construct in silencing genes. As the VA episome accumulate well in many varieties of plants, this episome is likely to function as a versatile VIGS vector in many plant species.

## Methods

### Construction of Viral Amplicon (VA) from ToLCV DNA-A

Several fragments of the viral DNA-A component as well as the 35S-Nos promoter-terminator region were cloned in the binary vector pCAMBIA1391Z in a serial manner as follows. A region containing *Cauliflower mosaic virus *35S promoter, multiple cloning site (MCS) and *nos *terminator was PCR amplified from *the *pRT100 vector (using the primers: Forward: 5'-AAGCTTAACATGGTGGAGCACGACACTC-3', and Reverse 5'-GAATTCGTCACTGGATTTTGGTTTTAG-3') and was cloned in the pCAMBIA1391Z vector at the *Hind*III - *Eco*RI site. Next, the CR-AC3 fragment of ToLCV (New Delhi isolate, [DQ629101]) was isolated through error-proof PCR using the DNA-A component of ToLCV as the template and the following primers: Forward: 5'-CACAAGCTTGTGATTGGTTAGCATGTCCT-3', and Reverse: 5'-TAAAAGCTTTTAATAAATATTGAATTTT-3'. The desired amplified PCR fragment was cloned in pGEMT and subsequently in pCAMBIA1391Z-35S-nos construct in sense orientation at the *Hind*III site. Finally, the common region (CR) region was amplified from ToLCV DNA-A template (using the primers: Forward: 5'-CACGAATTCGTGATTGGTTAGCATGTCCTTG-3', and Reverse: 5'-ATGGAATTCCATGTTGACTTAGTCAATCGG-3') and was cloned in sense orientation at the *Eco*RI site of the above-mentioned recombinant pCAMBIA1391Z. The final clone was designated as VA/pCAMBIA1391Z.

### Cloning of GFP gene in VA/pCAMBIA1391Z vector

The *GFP *gene was amplified by PCR using a template of *GFP *cloned in pGEMT (a kind gift from Dr. D. Baulcombe, USA) and the following primers: Forward: 5'-ATGAGCTCATGGCAAGTAAAGGAGAAGAAC-3', and Reverse: 5'-CGGGATCCGAGCTCTTAGAGTTCGTCGTGTTTG-3'. The amplified fragment was cloned in the forward orientation in VA/pCAMBIA1391Z, as obtained above, at *Sac*I sites. The resulting construct was designated as VIGS-*GFP*.

### Genomic DNA Isolation and Semi Quantitative PCR to check for episome formation

The genomic DNA was extracted from plant tissues by following the standard CTAB protocol [32]. Briefly, 5 g of plant tissues were crushed in liquid nitrogen and homogenized in a solution containing 5 M NaCl and 2% sarcosyl. The homogenized solution was centrifuged and the extraction buffer (100 mM Tris-Cl, pH 8.0, 20 mM EDTA, 1.4 M NaCl and 2% CTAB) was added to the supernatant. Following incubation at 60°C for 35 min, the solution was extracted with equal volume of CHCl_3_: Isoamyl alcohol (24:1 v/v). DNA was precipitated with isopropanol. Contaminating RNA was removed through treatment with RNase A (40 μg/ml) followed by phenol extraction. Purified DNA was dissolved in 10 mM Tris (pH 8.0), 1 mM EDTA and used for further manipulations. About 0.5 μg of DNA was used for PCR to check for episome (Viral Amplicon) formation using the following divergent primers: Forward: 5'-AAGCTTAACATGGTGGAGCACGACACTC-3', and Reverse: 5'-AGAAGCTTCTATGCGTCG TTGGCAGATTG-3' (designated as F and R respectively in Fig. 1B). The PCR was carried out for 27 cycles and actin gene was used as the internal control. The amplification products were separated on 1% agarose gels and the band intensities were quantified using Quantity One (BioRad, USA) software.

### Mutation of AC2 gene

A point mutation in *AC2 *gene was created by using Site Directed Mutagenesis kit (Stratagene, USA) with the following primers: Forward: 5'-GCGACCTTCGTAACCCTCGAAGGCCC-3', and Reverse: 5'-GGGCCTTCGAGGGTTACGAAGGTCGC-3', and CR-AC3 fragment cloned in pGEMT as the template. The protocol followed was as supplied by the manufacturer. The mutation resulted in the conversion of amino acid serine at position 5 to a stop codon in the AC2 ORF. The amplified PCR fragment was digested with *Hind*III and was used for cloning in pCAMBIA1391Z vector as described earlier to generate VA (*AC2M*) construct.

### Mutation of AC4 gene

A point mutation in *AC4 *gene was created by using Site Directed Mutagenesis kit (Stratagene, USA) as above, with the following primers: Forward: 5'-CTTGCATGTGCTAATCCAGTTCGAAGG-3', and Reverse: 5'-CCTTCGAACTGGATTAGCACATGCAAG-3' and CR-AC3 fragment cloned in pGEMT as the template. The mutation resulted in the conversion of amino acid serine at position 9 to a stop codon in the AC4 ORF. The amplified PCR fragment was digested with *Hind*III and was used for cloning in pCAMBIA1391Z vector to generate VA (*AC4M*) construct.

### Mutation of both AC2 and AC4 genes

The point mutations in both *AC2 *and *AC4 *were created by following the same method that used for creation of mutation of *AC4 *gene, as above, except that the template used was the CR-AC3 fragment cloned in pGEMT with *AC2 *mutation (as obtained above) as the template. The primers used were identical to those used for creation of the point mutation of *AC4 *gene. The amplified PCR fragment was digested with *Hind*III and was used for cloning in pCAMBIA1391Z vector to generate VA (*AC2MAC4M*) construct.

### Isolation of PCNA Fragment from Tomato

Total RNA was extracted from of Tomato (variety Pusa Ruby) and first strand cDNA made using the RevertAid H Minus First Strand cDNA Synthesis kit (Fermentas Life Sciences, USA) following manufacturer's protocol. PCNA fragment (300 bp) was amplified from it using the primers Forward: 5'-ACGGATCCGTTCTAGAATCGATTAAGGATCTGG-3', and Reverse: 5'-GGGGATCCCCATTAGCTTCATCTCAAAATCAG-3'. The primers were designed based on the sequence of *PCNA *of tomato [GenBank:AJ515747]. The amplified fragment was cloned in pGEMT vector (Promega, USA). PCNA fragment was excised out using *Bam*HI and recloned in the sense orientation in the VA/ToLCV (also referred to as VIGS) and VIGS (*AC2M*) constructs obtained above, at the *Bam*HI site of the MCS of the 35S cassette to generate VIGS-PCNA(*AC2N*) and VIGS-PCNA(*AC2M*), respectively.

### RT-PCR for Quantification of the PCNA gene

Total RNA was extracted from the infiltrated tomato leaves using TRIZOL reagent (Invitrogen, USA) and 3 μg was used as template for RT-PCR using the RevertAid H Minus First Strand cDNA Synthesis kit (Fermentas, USA). One microliter of the RT-PCR reaction was used to check for the transcript levels of the *PCNA *gene using the primers mentioned above. *ACTIN *gene was used as an internal control for RNA quantity in the RT-PCR. In all these cases the RT-PCR was carried out for 24 cycles. The PCR products were separated on a 1% agarose gel. The relative intensities of the bands were estimated by densitometric scanning using Typhoon 9210 scanner and analysed by ImageQuant TL software (GE Healthcare, USA).

### Detection of siRNA

Total RNA was extracted from the infiltrated tomato or tobacco leaves as above and size fractionated by the addition of 5% PEG, 0.5 M NaCl followed by centrifugation. Small RNA was precipitated by adding 3 volumes of ethanol to the supernatant as obtained above. The enriched small RNA was electrophoresed at 180 V on a 15% denaturing PAGE. A 21-mer oligonucleotide was used as a size marker. The gels were stained with ethidium bromide prior to transfer onto the membrane and used as loading controls. RNA was then transferred to Hybond N+ membrane (GE Healthcare, USA) by electroblotting in 1× TBE buffer at 0.32 mA.cm^-2 ^for 50 min. The probes (CR-AC3, *GFP *and PCNA) were prepared separately by PCR method using the CR-AC3 fragment, *GFP *gene and PCNA fragment cloned in pGEMT vector (30 ng) as templates respectively. The primers and the conditions of PCR used for the purpose were as mentioned above, except that the PCR reaction was carried out in presence of 50 μCi of [α-^32^P]dCTP (3000 Ci.mmol^-1^, Perkin Elmer Life Sciences, USA). The PCR product was purified through Sephadex G-25 matrix (GE Healthcare, USA) following the standard protocol. The hybridization using the specific probes was carried out at 35°C overnight followed by washing at 37°C with 1× SSC, 0.5% SDS twice for 30 min each. The membrane was dried and subjected to autoradiography. The relative intensities of the bands were estimated by densitometric scanning using Typhoon 9210 scanner and analyzed by ImageQuant TL software (GE Healthcare, USA).

### Plant growths

Tomato (*Lycopersicon esculentum*, variety Pusa Ruby) seeds were germinated in vermiculite and transferred to soil pots (two plants per pot) when the seedlings were 3 weeks old. They were acclimatized to pot condition for 1 week till the true leaves started appearing. The plants were grown at 25°C under 14 h light/10 h dark condition.

Tobacco (*Nicotiana xanthii*) seeds were grown under similar conditions as described for tomato, except that the seeds were grown in vermiculite for 4-5 weeks before being transferred to soil pots, where these were grown for further 6 weeks. *Arabidopsis thaliana *ecotype Columbia (col0) was grown in a growth chamber on vermiculite/Agro peat mix at 22°C, with a 16 h light/8 h dark cycle with ~62% humidity. Rice (*Oryza sativa*, variety Pusa Basmati) seeds were germinated in water soaked germination paper for 12 days. The seedlings were transferred to the soil pots grown for 3 weeks in a green house at 28°C, with a 16 h light/8 h dark cycle with ~85% humidity.

All the above experiments were carried out during the months of June through September.

### Agroinfiltration

*Agrobacterium tumefaciens *(strain LBA4404) was transformed with pCAMBIA1391Z, VA/ToLCV, VIGS-*GFP*, VIGS-PCNA(*AC2N*), VIGS-PCNA(*AC2M*), VIGS-PCNA(*AC4M*) and VIGS-PCNA(*AC2MAC4M*). The transformed cells were grown in Luria-Bertani (LB) medium containing 50 mg.L^-1 ^kanamycin and 25 mg.L^-1 ^streptomycin at 28°C in a gyratory shaker at 180 rpm for 24 h. The cells were harvested and resuspended in infiltration buffer (10 mM MgCl_2_, 10 mM MES, pH 5.6) to a final OD (600 nm) of 1.0. The culture was incubated in a gyratory shaker at 100 rpm for 3 h at room temperature and infiltrated (500 μl per infiltration) into the abaxial surface of the leaves of tomato, tobacco and *Arabidopsis *through 1 ml syringe. For infiltration into the rice leaves, the above culture (500 μl per infiltration) was harvested at 3000 rpm for 15 min and the cells were directly pricked into the leaves. The plants were allowed to grow under specified conditions and leaf samples were collected at desired time intervals.

## Competing interests

The authors declare that they have no competing interests.

## Authors' contributions

PP carried out most of the experiments. NRC helped in the Southern and Northern hybridization experiments, in quantifying and performing the statistical analyses of the data and in drafting the manuscript. SKM conceived of the study, and participated in its design and drafted the manuscript in its final form. All authors read and approved the final manuscript.
